# Norovirus infection among HIV-infected patients in Abuja, Nigeria: impact of combination antiretroviral therapy status

**DOI:** 10.1186/s12879-023-08592-3

**Published:** 2023-09-21

**Authors:** Favour Osazuwa, William Olayemi Johnson, Hailey Seth Grobler

**Affiliations:** 1 Department of Medical Laboratory Sciences, University of Benin, Benin City, Nigeria; 2 MDS Molecular Services, Sub-Saharan African Office, Abuja, Nigeria; 3 Laboratory of Molecular Virology, Biotech Africa Genomics, West African Region Office, Abuja, Nigeria

**Keywords:** Norovirus, cART, HIV, CD4+

## Abstract

**Background:**

Norovirus is now recognized to be major cause of gastroenteritis worldwide, with significantly higher disease burden among immunocompromised patients. This study aimed to determine the prevalence of Norovirus among HIV-infected patients and to evaluate the impact of combination antiretroviral therapy (cART) status on Norovirus prevalence in a sub-urban area of Abuja, Nigeria.

**Methods:**

This study included a total of Two hundred and fifteen subjects (85 cART-naïve and 130 cART-exposed) HIV-infected patients. Age range of study participants was 18 to 60 years. Faecal specimens where collected in screw capped containers and analyzed for Norovirus using Accupower Norovirus real-time PCR Test kit. CD4 + cell count was determined using flow cytometry.

**Results:**

The prevalence of Norovirus among cART-naïve HIV-infected patients was 10.6%. Age and gender was not associated with norovirus infection. cART –naïve HIV-infected patients with CD4 + cell count < 200 was significantly more infected with Norovirus as compared to those with CD4 + count ≥ 200 (OR: 28.000, 95% CI 3.2237, 243.2007, P = 0.0025). Norovirus was also found to be significantly higher in cART-naïve HIV-infected patients than amongst cART-exposed counterparts (OR: 6.882, 95% CI: 1.4514, 32.6343, P = 0.015).

**Conclusions:**

The prevalence of Norovirus among cART-naïve HIV-infected patients was high; and was significantly higher in subjects with low CD4 + counts. Screening for Norovirus among cART-naïve HIV-infected patients is however emphasized to allow for effective Norovirus disease management.

## Background

Norovirus continues to be a major etiologic agent of diarrhea worldwide, and it causes about 18% of all cases of acute gastroenteritis worldwide [1]. Norovirus also called the winter vomiting bug is characterized by non-bloody diarrhea, vomiting and stomach pain and in some cases dehydration [2]. Norovirus infection in severe cases results in norovirus-associated enteropathy, intestinal villous atrophy, and malabsorption [2].

Norovirus is capable of establishing a long term infection in people who are immunocompromised, such as those with common variable immunodeficiency, Human Immunodeficiency virus (HIV) or with a suppressed immune system after organ transplantation [3]. Characteristically, norovirus infection in HIV-infected patients lasts from weeks to years with concomitant difficulty in clinical management [4]. To the best of our knowledge, there is dearth of information on the proportion of norovirus infection among HIV-infected patients with diarrhea in Nigeria. This study thus aimed to determine the prevalence of Norovirus among HIV-infected patients in Abuja, Nigeria.

## Methods

### Study design and setting

This study was a one-year longitudinal cross-sectional study among HIV-infected patients presenting at three ART clinics in Yangoji, Abaji and Kwali communities of Abaji and Kwali Local Government areas of Abuja, Federal Capital Territory (FCT), Nigeria. This Study was carried out in the period February 2022 to January, 2023. The study area is mainly sub-urban, and it’s the farthest local area from the FCT city Centre.

#### Ethical approval

was received from Local health authority (HEC/LHA:130A5) before commencement of study. Within the study period, Eighty-Five (85) cART –naïve recently diagnosed HIV-infected patients both male and female with diarrhea were recruited after receiving verbal or written consent for inclusion. One hundred and thirty (130) cART exposed patients were also screened for Norovirus. Age range of study participants was 18 to 60 years.

### Sample collection and Processing

About 5 ml of blood was collected into EDTA vacutainer aseptically. Participants were tested for HIV using two different immunochromatographic methods: Determine 1 & 2 (Inverness Medical Japan Co, Ltd) and STAT-PAK (Chembio Diagnostic System, New York, USA) the tie breaker employed was Unigold HIV test kit.

Faecal specimens where collected by participants into well screw-capped universal containers and where safely transported to the laboratory within thirty minutes of collection. Screening for Norovirus was performed using AccuPower Norovirus Real-Time RT-PCR Kit (Bioneer, South Korea), an in vitro diagnostic kit designed for the detection of norovirus (GI, GII) RNA in human samples such as stool and rectal swab through real-time PCR. The PCR protocol was a one-step premix type with a vacuum dried premix. All PCR protocol was carried out on a Exicycler 96 PCR platform (Bioneer, South Korea). AccuPower Norovirus Real-Time RT-PCR Kit includes Positive Control (PC), No Template Control (NTC) and Internal Positive Control (IPC) for accurate and reliable diagnosis of norovirus. ExiDiagnosis software was used to analyze the test results based upon the Ct (threshold cycle) value. CD4 + cell counts were measured among cART naïve HIV-infected patients by flow cytometry (FACS scan, Beckton Dickinson, USA).

## Results

The overall prevalence of Norovirus among cART naïve HIV-infected patients in this study was 10.6% (9/85). Age and gender were not significant prevalence determinants of norovirus among study participants. cART-naïve patients with CD4 + counts < 200 were significantly more infected with norovirus as compared to subjects with CD4 + counts ≥ 200 (OR: 28.000, 95% CI 3.2237 to 243.2007, P = 0.0025) Table 1.

**Table 1:** Prevalence of Norovirus among cART Naïve HIV-infected patients

Parameter	Total Examined	Frequency (%)	OR	P value
**Age (yrs)**				
18-25	13	3 (23.1)		0.1420
26-35	25	6 (24.0)		
36-45	30	1 (3.3)		
≥ 46	17	1 (5.9)		
**Gender**				
Male	34	4(11.8)	1.2000	0.7963
Female	51	5 (9.8)		
**CD4+**				
<200	17	8 (47.0)		
≥200	68	1 (1.5)	28.0000	0.0025*

Norovirus was also found to be significantly higher in cART-naïve HIV-infected patients when compared to the cART exposed counterparts (Table 2).

**Table 2:** Proportion of Norovirus among cART naïve and cART exposed subjects

Parameter	Total Examined Frequency (%)		OR	95%CI	P value
**HAART-naïve**	85	9 (10.6)	6.882	1.4514, 32.6343	0.015*
**HAART-exposed**	130	2			

**Fig. 1:**
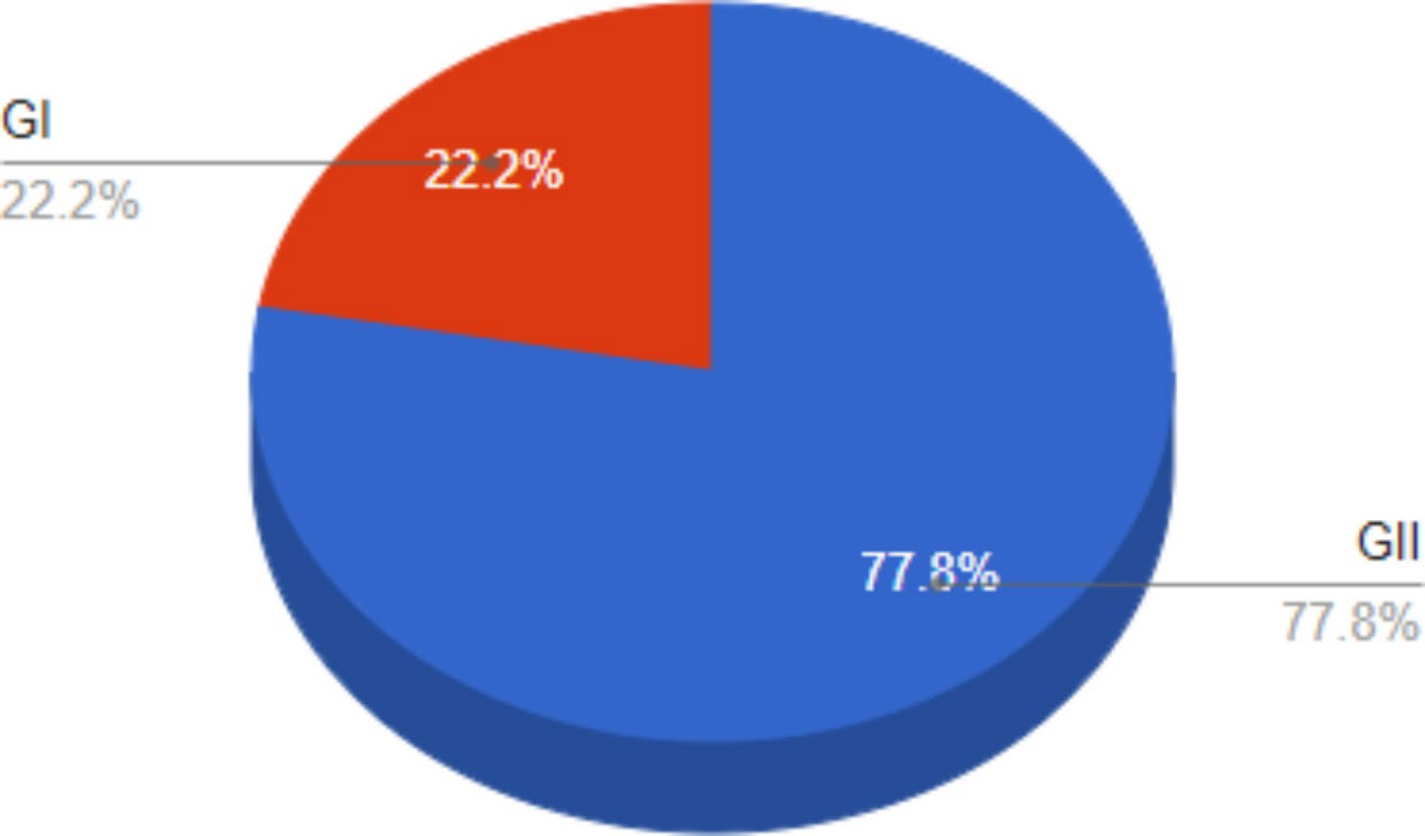
Norovirus genotypes detected among study participants

The most commonly encountered norovirus genotypes among cART-naïve patients in our study area was Nov GII (Fig. 1).

## Discussion

Norovirus is now recognized as the major cause of gastroenteritis world over [5]. Norovirus is known to have high prevalence among immunocompromised patients with concomitant chronic diarrhea rather than acute illness [6], our study reports a high prevalence of Norovirus among cART-naïve HIV-infected patients in Abuja, Nigeria. Our finding further provides strength to the growing body of knowledge incriminating Norovirus as one of the major infectious disease causing diarrhea in HIV-infected patients [7] and immunocompromised subjects [8].

Immunocompromised hosts have defective mechanisms to fight infections as compared to healthy individuals [9]. Immunocompromised hosts include patients with immunologic disorders, organ transplant recipients, patients on immunosuppressive therapies, cancer patients, patients with neutropenia and diabetic patients etc. [10]. Immunocompromised subjects are at higher odds of infection with Norovirus as compared to healthy individuals [11]. Norovirus infection in HIV-infected patients have been documented to produce more adverse outcomes than in non-HIV-infected patients [12]. Norovirus disease in HIV-infected patients causes profound and persistent diarrhea, with excessive weight loss [12].

A recent study identified Norovirus as the most common encountered viral enteropathogen among HIV-infected patients [13], further analysis showed that there was a significant association between Norovirus and onset and degree of diarrhea. Screening for Norovirus amongst HIV-infected patients is valuable, early detection and management is pivotal.

## Conclusion

Inclusion of norovirus screening in the work up for cART –naïve patients in our study area should be encouraged. Evaluation of immune status might provide better insights for patient segregation and care.

## Data Availability

The data and materials supporting the conclusions of the study are available from the corresponding author on reasonable request.

## Abbreviations

ART: Anti-retroviral therapy
cART: Combination anti-retroviral therapy
Nov: Norovirus
PCR: Polymerase chain reaction
HIV: Human Immunodeficiency Virus
OR: Odds Ratio
CI: Confidence Interval
CD4+: Cluster of Differentiation 4
EDTA: Ethylene diamine tetra acetic acid
